# 术前焦虑对胸腔镜肺癌根治术后患者早期预后的影响

**DOI:** 10.3779/j.issn.1009-3419.2019.11.10

**Published:** 2019-11-20

**Authors:** 云霄 张, 宗超 李, 冀衡 陈, 志毅 范

**Affiliations:** 100142 北京，北京大学肿瘤医院暨北京市肿瘤防治研究所麻醉科，恶性肿瘤发病机制及转化研究教育部重点实验室 Key Laboratory of Carcinogenesis and Translational Research (Ministry of Education/Beijing), Department of Anesthesiology, Peking University Cancer Hospital and Institute, Beijing 100142, China

**Keywords:** 焦虑, 肺肿瘤, 胸腔镜, 术后恢复, Anxiety, Lung neoplasms, Thoracoscopy, Postoperative recovery

## Abstract

**背景与目的:**

肺癌患者常伴焦虑，影响术后恢复。本研究旨在观察术前焦虑对胸腔镜肺癌根治术后患者早期预后的影响。

**方法:**

以胸腔镜肺癌根治术患者100例为研究对象，术前采用医院焦虑抑郁量表（hospital anxiety and depression scale, HADS）对患者进行评估，其中焦虑患者44例（焦虑评分≥8分）纳入焦虑组；非焦虑患者56例（焦虑评分 < 8分）纳入对照组，术后随访患者早期预后指标。主要研究终点：患者术后住院时间；次要研究终点：患者总住院时间、术后视觉模拟疼痛评分（visual analogue scale, VAS）、术后恶心呕吐及新发心律失常发生率、术后镇痛药和补救止吐药用量。

**结果:**

相对于对照组，焦虑组术后住院时间及总住院时间均明显延长[（5.1±2.5）d *vs*（4.0±1.3）d，*P* < 0.01；（10.9±4.0）d *vs*（9.1±4.1）d，*P* < 0.05]；术后VAS评分、恶心及新发心律失常发生率显著增高[（4.7±1.9）分*vs*（2.6±1.8）分，*P* < 0.001；40.9% *vs* 16.1%，*P* < 0.01；36.4% *vs* 20.7%，*P* < 0.05]；术后镇痛药和补救止吐药用量增多[(72.5±8.9) mL *vs* (68.2±9.4) mL, *P* < 0.05; (2.1±2.9) mg *vs* (0.9 ± 1.9) mg, *P* < 0.05]。

**结论:**

术前焦虑可影响胸腔镜肺癌根治术后患者早期预后，延长住院时间，增加患者术后VAS评分、恶心及新发心律失常发生率，增加术后镇痛药及补救止吐药用量。

## Abstract (en)

**Background and objective:**

Patients with lung cancer are often accompanied by anxiety, which affects postoperative recovery. The aim of this study is to explore the effects of preoperative anxiety on early prognosis in patients after thoracoscopic lung cancer resection.

**Methods:**

A total of 100 patients undergoing thoracoscopic resection of lung cancer were divided into 2 groups by hospital anxiety and depression scale (HADS): 44 in anxiety group (anxiety score > 8) and 56 in control group (anxiety score < 8). The primary endpoint: length of postoperative hospital stay. The secondary endpoint: length of hospital stay, visual analogue scale (VAS), the incidence of nausea and vomiting as well as postoperative new arrhythmia and the consumption of postoperative analgesic and rescue antiemetic.

**Results:**

Compared with the control group, the length of postoperative hospital stay and hospital stay in the anxiety group were both significantly longer [(5.1±2.5) d *vs* (4.0±1.3) d, *P* < 0.01; (10.9±4.0) d *vs* (9.1±4.1) d, *P* < 0.05)], the VAS score and the incidence of nausea as well as arrhythmia were significantly increased [(4.7±1.9) *vs* (2.6±1.8), *P* < 0.001; 40.9% *vs* 16.1%, *P* < 0.01; 36.4% *vs* 20.7%, *P* < 0.05], and the consumption of postoperative analgesic and rescue antiemetic were also significantly increased [(72.5±8.9) mL *vs* (68.2±9.4) mL, *P* < 0.05; (2.1±2.9) mg *vs* (0.9±1.9) mg, *P* < 0.05].

**Conclusion:**

Preoperative anxiety can affect the early prognosis of patients after thoracoscopic lung cancer resection, prolong hospitalization time, increase the postoperative pain score and the incidence of postoperative nausea and new arrhythmia as well as the consumption of postoperative analgesic and rescue antiemetic.

肿瘤患者常伴有焦虑，发生率可达10%-50%，其中以肺癌、妇科肿瘤患者焦虑发生率最高^[1-4]^。有研究^[5, 6]^报道焦虑可影响肿瘤患者的生活质量、病情进展甚至患者长期生存，但关于焦虑对肿瘤患者术后早期预后影响的研究较少，本研究旨在探索术前焦虑对胸腔镜肺癌根治术后患者早期预后的影响。

## 1 资料与方法

### 1.1 研究对象

选择2017年8月-2018年8月于北京大学肿瘤医院行胸腔镜肺癌根治术的100例患者为研究对象，患者及其家属签署知情同意书。纳入标准：年龄18岁-70岁，体质指数18 kg/m^2^-25 kg/m^2^，美国麻醉师协会（American Society of Anesthesiologists, ASA）分级Ⅰ级-Ⅱ级。排除标准为：认知障碍患者，既往慢性疼痛史，有镇痛药、镇静药或抗抑郁药长期使用史及妊娠、非哺乳期患者。本研究采用了改编的中文版“简易精神状态检查量表（Mini-mental State Examination, MMSE）”识别认知障碍患者：MMSE是临床常用的神经心理学检查工具，能较全面快速地评估被测试者智力状态及认知功能下降程度^[7]^，量表共30项题，内容涉及时间定向力、地点定向力、即刻记忆、注意力及计算力、延迟记忆、语言、视空间7个方面，每项回答正确得1分，回答错误或答不知道评0分，量表总分范围为0分-30分。术前24 h对患者进行量表评估，截止点如下：文盲患者至少得13分；学历1年-7年的患者至少得18分；学历8年或以上的患者至少得26分，评分低于截止点者排除在研究之外。

### 1.2 研究方法

#### 1.2.1 患者分组

手术前24 h由专人采用医院焦虑抑郁量表（hospital anxiety and depression scale, HADS）对纳入的患者进行焦虑状态评估。HADS是筛查焦虑抑郁状态的最常用工具之一^[8]^，其中焦虑量表由7个评估焦虑状态的问题组成，每个问题用4个分值来反映躯体和心理症状，答案的数值范围从0-3，其总和可以从0分-21分。最终评分 < 8分说明无症状，8分-10分为可疑症状，11分-21分则说明肯定存在焦虑状态，实际评分中以8分为起点，即可疑和有症状者均认为是阳性，纳入焦虑组， < 8分者进入对照组^[9]^。

#### 1.2.2 麻醉方法

患者入室后给予常规监测，建立静脉通路。静脉注射丙泊酚2 mg/kg-3 mg/kg、舒芬太尼0.02 µg/kg、顺式阿曲库铵0.2 mg/kg进行麻醉诱导并气管插管，之后连接呼吸机行机械通气，维持P_ET_CO_2_ 30 mmHg-40 mmHg。七氟醚吸入复合瑞芬太尼静脉输注进行麻醉维持，每间隔30 min静脉注射顺式阿曲库铵0.2 mg/kg，维持脑电双频指数（bispect ral index, BIS）值于40-60，血压和心率波动幅度不超过基础水平的20%。手术结束前30 min停用肌松药和瑞芬太尼并给予喷他佐辛0.8 mg/kg止痛。手术结束时，停止吸入七氟醚，并静脉给予阿托品1 mg和新斯的明2 mg拮抗肌松残余效应，待患者呼吸恢复满意后拔除气管导管。

拔管后每5 min对患者进行一次疼痛评分，当疼痛程度采用视觉模拟量表（visual analog scale, VAS）评分≥4分时，静脉注射枸橼酸舒芬太尼注射液（批号：180265，Eurocept BV公司，荷兰）5 µg，必要时间隔5 min重复给药，直至患者VAS评分≤3分，随后开启Accufuser Plus P2015MD型镇痛泵（江苏爱朋医疗，中国）行患者自控静脉镇痛（patient controlled intravenous analgesia, PCIA）。PCIA泵药液配方均为舒芬太尼注射液250 µg、托烷司琼30 mg加入生理盐水至100 mL。参数设定为预充量2 mL，PCA 2 mL，背景输注速率1 mL/h，锁定时间10 min，患者苏醒完全后送回病房。患者完全依靠PCIA行术后镇痛，若有恶心呕吐发生，可给予盐酸托烷司琼注射液5 mg/次。

#### 1.2.3 评价指标

主要评价指标为患者术后住院时间，次要评价指标为患者总住院时间、术后VAS评分、恶心呕吐发生率及新发心律失常发生率、术后镇痛药用量和补救止吐药用量。

### 1.3 统计学处理

采用SPSS 22.0进行统计分析。连续变量采用*t*检验方法对主要终点进行分析，数据以均数±标准差表示。分类变量采用χ^2^检验进行分析。*P* < 0.05（双侧）为差异有统计学意义。

## 2 结果

### 2.1 两组患者人口学资料和一般情况比较

研究共招募患者106例，其中2例患者拒绝参加研究，4例患者存在认知障碍，最终入组患者100例。HADS评分显示，100例患者中，44例患者焦虑评分≥8分，进入焦虑组，56例患者焦虑评分 < 8分，进入对照组。两组患者人口学资料和一般情况比较无统计学差异（*P* > 0.05），见表 1。

**1:** 两组患者人口学资料和一般情况 Demographic and baseline characteristics

Variable	Control group (*n*=56)	Anxiety group (*n*=44)	*P*
Age (yr)	58.1±7.3	58.7±6.9	0.697
Gender (Male/Female)	24/32	19/25	> 0.999
Weight (kg)	64.1±9.2	66.2±10.9	0.293
Height (cm)	162.7±7.8	163.6±8.0	0.562
ASA class Ⅰ/Ⅱ	6/50	4/40	> 0.999
Comorbidities			
Hypertention	17	13	> 0.999
Diabetes	3	1	0.628
Coronary heart disease	6	2	0.460
Arrhythmia	2	2	> 0.999
Hepatic dysfunction	9	3	0.219
Renal dysfunction	3	2	> 0.999
Type of surgery			0.841
Right VATS lobectomy	30	22	
Left VATS lobectomy	26	22	
Surgeon			0.202
A	35	33	
B	21	11	
Duration of surgery (min)	110.6±30.6	103.3±25.8	0.205
Estimated haemorrhage (mL)	74.5±46.2	98.9±118.1	0.160
Data are expressed in Mean±SD or number. ASA: American Society of Anesthesiologists; VATS: video-assisted thoracoscopic surgery.			

### 2.2 住院时间比较

与对照组患者相比，焦虑组患者术后住院时间显著延长（*P* < 0.01）；焦虑组患者总住院时间明显延长（*P* < 0.05），见表 2。

**2:** 两组患者术后早期预后 Early prognosis between the two groups

Variable	Control group (*n*=56)	Anxiety group (*n*=44)	*P*
Length of hospital stay (d)	9.1±4.1	10.9±4.0	0.038
Preoperative preparation time (d)	5.3±3.7	5.7±3.9	0.612
Postoperative hospital stay (d)	4.0±1.3	5.1±2.5	0.004
Visual analog scale	2.6±1.8	4.7±1.9	< 0.001
Nausea [*n* (%)]	9 (16.1)	18 (40.9)	0.007
Vomiting [*n* (%)]	5 (8.9)	7 (15.9)	0.358
New arrhythmia [*n* (%)]	9 (16.1)	16 (36.4)	0.035
Postoperative analgesic consumption (sufentanil, mL)	68.2±9.4	72.5±8.9	0.020
Postoperative rescue antiemetic consumption (tropisetron hydrochloride, mg)	0.9±1.9	2.1±2.9	0.027
Data are expressed in Mean±SD or number (%); New arrhythmia included postoperatively occured tachycardia and atrial fibrillation.			

### 2.3 术后疼痛比较

与对照组患者相比，焦虑组患者术后VAS疼痛评分显著增高（*P* < 0.001），术后镇痛药用量增加（*P* < 0.05）。

### 2.4 术后恶心呕吐及新发心律失常比较

与对照组患者相比，焦虑组患者术后恶心发生率显著升高（*P* < 0.01），术后补救止吐药用量增加（*P* < 0.05）；焦虑组患者术后新发心律失常发生率明显升高（*P* < 0.05）；两组患者术后呕吐发生率无显著差异（*P* > 0.05），见表 2。

## 3 讨论

本研究以胸腔镜肺癌根治手术患者100例为研究对象，采用HADS将纳入研究的患者分为焦虑组44例和对照组56例，结果显示，与对照组比较，术前焦虑患者术后住院时间及总住院时间明显延长，术后VAS评分、恶心发生率及新发心律失常发生率显著增高。

肺部肿瘤患者常伴有焦虑^[10]^，有研究^[11]^表明其发生率可达50%，居各类肿瘤患者前列。原因可能涉及患者对于手术的严重恐惧、术后疼痛、术后并发症及物理功能障碍等方面，本研究纳入拟行胸腔镜肺癌根治术患者100例，术前采用HADS对其进行评估，结果发现焦虑患者（焦虑评分≥8分）44例，发生率为44.0%，总体发生率与相关报道近似。

为了评估术前焦虑对患者术后总体恢复质量的影响，本研究将术后住院时间作为主要研究终点，结果显示，相对于对照组患者，焦虑组患者术后住院时间和总住院时间均明显延长。与此结果一致，一项回顾性研究^[12]^也表明，术前焦虑的患者伴随着住院时间的延长，而术前服用抗焦虑药可以缩短术后住院时间；另一项关于腰部术后患者的研究^[13]^也表明术前焦虑是延长患者住院时间的独立危险因素。

焦虑患者对于疼痛更加敏感，一项针对于开腹肾癌切除术患者的研究^[14]^表明，术前焦虑会加重患者术后疼痛程度，而且在术后早期（12 h-24 h）表现最为明显；另一项涉及乳腺癌切除术、开胸肺癌切除手术等9种手术方式在内的涵盖3, 112例患者的回顾性研究^[15]^发现，术前焦虑评分高的患者术后疼痛评分更高，使用镇痛药量更大，而且更易于转化为慢性疼痛。影响术后术后疼痛的因素很多，包括遗传因素、疼痛敏感性、情绪、手术类型、持续时间和镇痛类型^[16, 17]^，本研究中两组患者的手术类型、手术持续时间和术后镇痛类型均无明显差异，结果显示焦虑组患者术后24 h及48 h镇痛评分显著高于对照组患者，术后镇痛药用量增加。

本研究还发现，与对照组相比，焦虑组患者术后恶心发生率升高，术后补救止吐药用量增加。与此相近，一项纳入了1, 389例患者的回顾性研究^[18]^表明，术前焦虑是患者术后恶心呕吐发生的独立预测因素。另有研究^[19]^表明术前焦虑与患者术后早期（12 h）恶心呕吐发生率高有关。

本研究观察了焦虑与肺癌患者术后心律失常发生之间的关系，结果显示，术前焦虑组患者术后窦性心动过速和房颤发生率显著高于对照组。心律失常特别是房颤，是肺部切除术后常见并发症，发生率可达12.9%，常发生于术后2 d-3 d，可能原因在于肺部切除后右心房容量负荷增大及术后低氧有关^[20]^。而焦虑与房颤的关系在之前研究中已有发现，有研究^[21]^表明，焦虑患者更易伴发房颤。另有一项涉及378例患者的研究^[22]^进一步证实焦虑与房颤加重亦有关联。

本研究采用了HADS对患者进行焦虑状态评估，HADS量表是对住院患者进行焦虑抑郁状态评估的常用方法^[23]^。另外本研究入组患者中术后并发症发生率较低，因为本研究只分析了焦虑对胸腔镜肺癌切除术后患者心律失常的影响，在以后的研究中我们会进一步分析焦虑对肺癌切除术患者其他术后并发症的影响。本研究证实术前焦虑可影响肺癌根治术后患者早期预后，而如何缓解患者术前焦虑并由此改善患者预后将是我们今后研究的重点。

综上所述，术前焦虑可影响胸腔镜肺癌切除患者术后早期预后，延长住院时间，增加患者术后疼痛评分及术后心律失常发生率。
